# Mr. Clarke on Those Diseases of Females Attended by Discharge

**Published:** 1822-06-01

**Authors:** 


*Observations on those Diseases of Females which are attended by Discharges. Illustrated by Copper-plates of the Discharges.* By Charles Mansfield Clarke, Member of the Royal College of Surgeons, London. Part II. 8vo. pp. 242. London, 1821.

[Second Analytical Article, continued from No. 8, p. 778, Vol. II.]

To those who have properly considered the nature and objects of this Journal, it will be unnecessary to give any reason why our analyses are sometimes carried to an unusual length, as compared with similar articles in other periodicals. It is quite out of the question that we can compete with our swift-footed monthly cotemporaries, in bringing the *earliest samples* of medical lore into the literary market. It is the object and office of these *avant-couriers* to whet the appetites of the reading public—it is ours to lay more substantial fare before them—and to constitute a medium between the ponderous octavo itself, and the multiform critiques that "spread their light wings," and waft its condemnation or praises through the medical republic. It will always be our anxious wish to proportion our reviews to the value and importance of the works reviewed, though we cannot hope, in so doing, to give entire satisfaction to all parties—a consummation that, however devoutly it is to be wished, can never be rationally expected in this sublunary scene. We shall do what we think best, and appeal to old Chronos for judgment.

In our first analysis of Mr. Clarke's work, we concluded the subject of "Watery Discharge." We now come to the third and last chapter, which treats of—"the Purulent Discharge." The characters of this, are "a heavy, yellowish, opake fluid, possessing little tenacity." Though small in quantity, as compared with the watery discharge, it is far more exhausting to the constitution. Pus may be secreted from membranes without breach of surface, and is then unmixed with blood, which appears, after any violence, where the discharge is from an ulcerated surface—a tolerably fair criterion, Mr. C. thinks, as to whether the pus is secreted by a membrane in a state of inflammation or ulceration. But as a nice distinction of these cases is not always easy, our author first points out those cases of purulent discharge, appearing to arise from the female mucous membranes in a state of inflammation, and, secondly, those other cases, where the pus proceeds from an ulcerated surface.

The mucous membranes lining the vagina, the uterus, and the fallopian tubes, are very differently affected during inflammation. In the two latter, coagulating lymph is generally extravasated, when the inflammatory action runs high. In painful menstruation, (inflammation of the mucous membrane of the uterus) flakes of coagulating lymph are almost always thrown off—in some cases forming accurate casts of the cavity; and this has happened in the fallopian tube also. Sometimes, however, the uterine mucous membrane will secrete pus, and this being discharged *per vaginam*, will render it doubtful whence it proceeds. At other times, the purulent secretion will be retained in utero, in consequence of adhesive inflammation of the cervix uteri. Coagulable lymph is rarely the product of vaginal inflammation—pus is produced by a very slight phlogosis there.

I. *Purulent Secretion from the Uterine Lining.* When this finds its way readily into the vagina, there will be few other symptoms than a sense of heat and uneasiness in the passages. In some cases, where the vagina is wholly free from inflammation, the patient experiences acute pain in the back and bottom of the abdomen, which being severe and constant, the practitioner examines, and finds the uterus tender to the touch, its size being also increased, resembling the viscus when impregnated. The uterus enlarging, the case is still doubtful, till, suddenly, a large quantity of offensive pus escapes, and relief immediately follows. It is no wonder that an unfavourable prognosis is sometimes made in these cases, the practitioner fearing (and with reason) that some morbid alteration of structure is taking place in the uterus. Two or three cases are here related in illustration. In one case, the uterus burst and discharged the matter into the abdomen, death being the necessary result. In the other case, the matter found its way into the rectum, and was voided by stool, with ultimate recovery.

*Treatment.* The symptoms will naturally point to the proper treatment—which is that employed in removing inflammation. Cup the sacral region—or apply leeches to the groins once a week—use the hip-bath night and morning—throw warm water up the vagina—give opium or other narcotics to diminish sensibility, while purgatives are to be employed to prevent constipation, and lessen inflammatory action. If the uterus attain the size of the fourth month of pregnancy, it may be presumed that the disease is *not* carcinoma—no tumour of this kind having ever attained such a size in our author's experience. The rapidity of purulent enlargement of the uterus, comparatively with that of fleshy tubercle, will throw some light on the diagnosis; and if there be reason to suppose the enlargement depends on purulent accumulation, "it may be advisable, gently to introduce the extremity of a bougie, or of a male catheter, into the os uteri, and to pass it onwards, until it has reached the cavity of the uterus."

II. *Vaginal Inflammation.* The symptoms attending common and specific inflammation of the vaginal membrane are nearly the same, and are pretty well known. Attempts indeed, have been made to distinguish between the purulent discharge of gonorrhœa and of common inflammation; but such pretensions are generally professed for self-interested purposes. The lymphatic glands of the groin, Mr. Clarke thinks, are more disposed to enlarge and suppurate in gonorrhœal, than common inflammation.

"In simple inflammation of the mucous membrane of the vagina, the purulent discharge being established in large quantity, the inflammatory symptoms frequently subside very rapidly, after which a termination is put to the secretion; the parts returning to a state of health, provided there be no acting cause producing its continuance; in which case the symptoms will continue until its removal, when they will speedily cease.

"In the case arising from specific contagion, the duration of the disease is greater; and the discharge, once established, continues for weeks, or perhaps for months, although not always accompanied by the other local symptoms." 169.

Mr. C. justly remarks that, it is impossible to determine at what period of the disease, the power of communicating infection ceases. A prudent practitioner will be careful to give no decisive opinion on the subject, especially as regards the non-existence of this power—for, as Mr. Clarke properly observes, "no person is secure from danger, who indulges in intercourse with a woman so long as the discharge continues."

"It is a curious fact, that in young subjects, both male and female, purulent discharge from the urethra and from the vagina takes place in consequence of the existence of irritation in distant parts; thus, during dentition, whilst the capsule of the tooth, or the gum covering it, is violently pressed upon by the crown of the tooth, the above circumstance is not unusual; medical men, therefore, should be careful to avoid denominating this symptom venereal; since, were it actually so, it would lead to nothing useful in the treatment; and discussions, highly destructive of the peace of families, might be thereby introduced." 170.

Mr. Clarke leaves, of course, the consideration of gonorrhœal inflammation to those writers who have made it the subject of distinct monographs; but, he gives it as his opinion, en passant, that the treatment of venereal gonorrhœa, differs little, if at all, from that which is applicable to common inflammation. As far as temperance, rest, and antiphlogistic measures are concerned, this may be true; but, would bals. copaiba and cubebs be applicable to *common* inflammation of the lining membrane of the urethra?—We should not like to try the experiment.

When the inflammatory symptoms have subsided, and a purulent discharge continues from relaxation or mal-habit of secretion, then our author recommends the cinchona, and copaiva; with astringent injections. Respecting the possibility of secondary symptoms following gonorrhœa, our author seems undecided— at least, he declines to offer an opinion upon the subject.

" He has certainly seen copper-coloured spots on the bodies of patients, who have laboured under gonorrhœa virulenta, removable only by the oxymuriate of mercury and sarsaparilla: but he thinks that he has seen similar appearances upon the skin of patients, whose chastity could not be suspected." 173.

Mr. Clarke has known several instances of married women, who had laboured under a purulent discharge, (possibly the effect of contagion,) bringing forth children prematurely, some dead, and others having dark-coloured furfuraceous cuticle in different parts of the body, which yielded only to mild preparations of mercury.

Modern experience has put the matter quite beyond a doubt, that secondary symptoms will occasionally follow gonorrhœa; but the chances are so much against the accident, that we should never dream of giving mercury as a preventive. Indeed, we are disposed to believe that, were mercury given as a preventive, there would be more secondary symptoms than if omitted, in consequence of imprudence in diet, and exposure to wet or cold, which, it is well known, are very injurious where mercury is in administration.

Abscesses sometimes occur in the cellular membrane surrounding the vagina, and do not admit of the same modes of cure, that are applicable to other purulent discharges. Such cases, however, are very rare. At the commencement of the complaint, there is nothing to discriminate it. Inflammatory symptoms are followed by a discharge of matter, and the patient supposes herself well. But after some time, a sense of fulness and pressure is experienced, and pus again escapes. On examination, a soft tumour will be found behind the vagina, which, being pressed, discharges pus. A continuance of this state impairs the health, and the neighbouring parts become more than usually irritable. Indeed, it has only occurred in women of lax fibre and irritable constitution. In cases of this kind, the abscess generally breaks into the vagina high up, which aggravates the inconvenience, the abscess being seldom emptied of its contents, which become, from retention, highly offensive. These cases are very unmanageable. No astringents can restrain the discharge—no stimulants can reach the cavity of the abscess—and it is hazardous to attempt an operation, unless the most depending part of the abscess should be situated so low, as to be capable of being brought within sight of the surgeon. Mr. Clarke relates three cases. In two of them, there was a cure effected by remedies adapted to improve the general health. In the third, he failed to remove the diseases.

III. *Ulceration of the Os and Cervix Uteri.* Our author has long been in the habit of describing two different kinds of ulceration of the uterus—the one denominated the "corroding," the other, the "carcinomatous" ulcer of the os uteri. They are both malign.

*Corroding Ulcer.* This disease usually takes place about the cessation of the catamenia. About this period, the uterus naturally attains a larger size than ordinary, and is not reduced to its usual volume, until the balance of the constitution is restored.

Inflammation attacking a part of firm texture, as the os uteri, an extravasation of coagulable lymph is more frequently formed than abscess, occasioning a thickening of the part. Sometimes this proceeds to ulceration—sometimes the inflammation recedes, leaving only an induration of the part.

"In the corroding ulcer of the os uteri, the membrane which covers it first takes on disease, and very shortly afterwards the ulcer extends to the whole circumference of the opening, and to the parts immediately beneath it; so that the natural shape of the os uteri is destroyed. Thence the ulceration proceeds to the cervix, and consumes it; so that, if the patient should die in this stage of the disease, nothing will be found, after death, but the body and the fundus of the uterus. Sometimes the disease does not stop here, but, before the patient is destroyed, the absorbents employed in the process of ulceration will have taken up nearly the whole body of the uterus, so that very little more than the fundus will remain." 188.

This does not happen in the carcinomatous ulcer, by which the patient is worn down before there is time for such a degree of absorption to take place. "If an examination be made, per vaginam, the breach of surface may be readily distinguished, and the extent of the disease ascertained; but no hardness of the parts will be present, no thickening, no deposit of new matter." The appearances, post mortem, will correspond. From our excellent author's symptomatology, we shall make the following extract.

"The menstruous secretion, it has been already said, has ceased; in its stead a yellowish discharge escapes, perhaps trifling in quantity, and now and then mixed with a streak of blood; by degrees the sense of warmth is converted into a glowing heat, affecting the region of the uterus; and it is by no means uncommon with patients to state, that they feel 'as if a hot coal was within them.'

"As this sensation of heat increases, so the quantity of the discharge increases, the ulceration extending more rapidly.

"The perpetual drain necessarily diminishes the quantity of circulating blood; in consequence of which the countenance becomes pallid, and weakness of the whole system is produced." 191.

Had the disease been carcinomatous ulceration, lancinating pain would have been the most prominent feature of the complaint. But this distressing symptom is comparatively absent in the ulceration under discussion.

"When a finger, introduced into the vagina, is made to pass over the ulceration, the patient does not complain of pain; she does not suddenly shrink from pressure, as when carcinomatous ulceration is present: but if asked what sensation she experiences, she will commonly reply, that she has a sense of soreness." 192.

Still the disease in question is as uniformly fatal as carcinoma uteri; but it will last during a much longer time, unless the fatal termination is hastened by hæmorrhage.

*Treatment.* Although the uterus performs no office in the constitution when menstruation has ceased, yet it is liable to morbid changes, that too often involve the constitution in destruction. Ulceration having once commenced in the uterus, this organ never, in our author's experience, recovers its healthy structure; "but increased action of the blood-vessels of the os uteri, which would eventually terminate in ulceration, may probably be diminished or controlled, so that no ulceration may take place, and, by such a mode of treatment, much advantage may be gained."

"Whenever, therefore, a patient in whom the menstruous secretion has recently ceased, complains of an increase of heat in the lower part of the back, or of the abdomen, or in the parts of generation themselves, a prudent practitioner, foreseeing the probable result, will direct the loss of some blood from the neighbouring parts. The most precise mode of obtaining this blood will be by cupping; although, if the patient be averse to the operation, leeches may be applied; but, upon the whole, they do not afford the same certain and immediate relief, neither can the quantity of blood removed by them be so exactly estimated." 194.

The operation should be repeated at the termination of a fortnight or three weeks, and if the sense of heat should continue, a still further loss should be directed—since temporary weakness is the only disadvantage which can accrue. General bleedings are not proper of course. The warm hip-bath twice a day is highly serviceable, and some of the warm water may be thrown up the vagina—both processes are equally beneficial in the indurated and ulcerated stages of the complaint.

"Saline purgatives, exhibited in very small doses, possess not only the power of allaying inflammation, by the watery secretions which they produce from the intestines, but they appear also to possess a specific power of tranquillizing the system, when in a state of disturbance and increased action, even when they produce very little sensible effect." 196.

Small doses of the sulphas magnesiæ vel potassæ may be exhibited twice a day, in combination with moderate doses of conium or hyoscyamus. Mr. Clarke does not object to the use of sarsaparilla or other alterative, provided such medicines do not derange the functions of the stomach, or impair the powers of the constitution. An abstemious diet should be enjoined, as fish, puddings, boiled fruits, and vegetables; and all excitants religiously avoided.

Even in the second stage, when ulceration is proceeding, and the patient is already weakened by the purulent discharge, what supports that ulceration but the inflammatory process? What remedies are likely to be more serviceable than those which retard it? It at length, however, happens that the patient becomes so debilitated by the purulent secretion that she is in danger of sinking from its effects. Mild astringent injections must then be thrown up into the vagina. Hæmorrhage may arise, and then still stronger applications are necessary, as alum in decoct. querci vel granati. If this should fail, solutions of sulphate of copper, or even nitrate of silver must be employed. The horizontal position—and small doses of the acidum sulphuricum in a mixture containing equal parts of decoct. cinchonæ and infus. rosæ, will be found proper auxiliaries.

IV. *Ulcerated Carcinoma of the Rectum.* The vicinity and sympathy between the rectum and uterus, will render it sometimes difficult to determine which part is affected, without an accurate enquiry and examination. The uterus is *more* liable to cancer than the rectum—and this should be borne in mind. In the early stage of carcinoma recti there is a mucous discharge, which gradually becomes purulent, and this appearance may lead us to suspect fistula, but examination puts the question to rest.

"If the finger of the practioner be carried into the rectum, it will be girt by a constriction of considerable thickness, through which it cannot be passed; and if any attempt is made to surmount the difficulty by violence, the patient will suffer excruciating pain, and a discharge of blood will be the consequence of such a rude enquiry." F. 203.

It is useless to be too anxious to ascertain the extent of the disease. A small carcinomatous thickening of the intestinal canal is as fatal as that of a larger portion. A carcinoma affecting not more than a quarter of an inch of the rectum may, by obstructing the passage of the fæces, cause a distension of the whole colon, and fatal inflammation, the consequence of that distension—a carcinoma of great extent can do no more.

In *common* ulceration the part is absorbed—in *carcinomatous*, new matter is deposited, as the old is removed, and thus the thickening and destructive processes proceed simultaneously. It is evident that the rectum cannot be kept at rest; and if the constipation be not viewed in relation to its proper cause, and the patient be exposed to the action of frequent purgatives, the symptoms will be aggravated by the means employed to alleviate them. The following melancholy picture is not overcharged, as we have too often had opportunities of verifying its correctness.

"All the symptoms which attend the first stage of this disease will be found to exist in a greater degree in the second. The darting pain will be increased both in frequency and in violence; the action of the heart will be greatly and permanently accelerated; the functions of the stomach will become more and more impaired; vomiting will be almost constantly present; temporaiy relief will be found only in opium; and permanent rest. only in the grave. In the progress of the ulceration, blood-vessels will be exposed which will pour out, according to their size, a larger or a smaller quantity of blood: and happy would it be for the patient if such hæmorrhage should prove fatal; but such an event is hardly to be expected; and, unless in parts more abundantly supplied with blood than the rectum, such an occurrence is seldom met with." 207.

As nearly the same treatment is necessary in this case as in ulcerated carcinoma of the uterus, we shall proceed at once to the latter subject.

V. *Ulcerated Carcinoma Uteri.* The carcinomatous *tumour* of the cervix uteri has been treated of in our author's former volume, to which we must refer the reader. A woman may live many years under such circumstances, provided she submits to proper rules; but sometimes our best efforts fail, and the ulcerative process takes place, on a more or less extended surface. In the latter case, the disease will be tedious—in the *former*, the fatal termination will be rapid. It ought to be borne in mind, however, that the ulceration goes on more rapidly at first, than in the progress of the disease—owing probably to weakened power of absorption in the latter period, keeping a ratio with the general decline of the powers of life.

"Thus a number of instances will be found, in which the patient will exist in a state of extreme weakness during many weeks or even months, contrary to the expectations of the medical attendant. Spontaneous bleedings from the ulcerated surface, producing more sudden debility, will have the same effect in retarding the progress of the disorder." 212.

In an early stage of the ulceration, it is not unusual for the patient to complain of a puffy and enlarged state of the external organs—owing to the increased action of the neighbouring vessels. A great degree of itching is another symptom, and sometimes erysipelas. The cuticle often desquamates—trifling oozing ensues, which, drying on the surface, forms furfuraceous scales, a new source of irritation, often excending to the groins and insides of the thighs. The discharge, at first ichorous, and afterwards purulent, is by no means comparable in quantity with that which is met with in some other diseases of those organs—and sometimes even diminishes as the disease advances, in consequence of the diminished quantity of blood in circulation.

If the bladder and rectum have not suffered in the early stage, they seldom escape now—not only from sympathy, but from the disease extending itself to these, in common with the adjacent parts. Such a degree of thickening takes place sometimes in the meatus urinarius as to impede the passage of the urine, and require the catheter—shortly after which the urine will spontaneously escape, not through its urinary passage, but through a communication between the neck of the bladder and vagina.

In a very few cases a communication between the rectum and vagina takes place, and from that moment no fæces pass by the anus—the external parts forming the channel through which urine, fæces, and pus are discharged. The stench now becomes intolerable, and the hips of the patient lying immersed in the excreted matters, the soft parts inflame, and sloughing takes place! It is needless to say that the wear and tear of the constitution, under such circumstances, is great. The patient becomes a skeleton, and dies under a complication of the most terrible phenomena which it is the lot of hapless mortality to endure! The situation of a woman labouring under carcinoma uteri is infinitely more pitiable than that of one with cancer in the breast; for not only are the symptoms more numerous and insufferable, but she has not the good fortune to be cut off, in the progress of the disease, by accidental occurrences. In mammary carcinoma the patient is usually destroyed by hydrothorax—but no such blessing is afforded to the subject of carcinoma uteri; the sufferer being compelled to endure till her frame is exhausted by pain, by vomiting, by want of sleep, by discharge, by an offensive atmosphere, or by gangrene of the integuments!

VI. *Treatment.* Though carcinomatous ulceration does not obey the laws by which common inflammation is governed, it is nevertheless controlled, in some measure, by those means which subdue common inflammation.

"Whenever a patient, labouring under carcinoma of the uterus, has placed herself under the constant care of a physician or surgeon, it will be necessary that he should watch with attention the changes which take place in her constitution. If he should find the circulation becoming accelerated, the skin more than usually hot, flabbiness of the integuments, softness and shrinking of the muscles in different parts of the body, he may presume that some important change has taken place in the diseased organ. If, together with these symptoms, the lancinating pain has been rendered more acute; if the sympathies between the uterus and the adjacent parts, or between that organ and the stomach, have been more than usually called forth; or if, lastly, the mucous discharge has assumed a puriform character, there can be little doubt that a breach of surface has taken place, and that the complaint has acquired its most frightful and distressing character." 219.

If the patient possess a vigorous constitution, we are hardly authorized to omit general bleeding; but this will seldom be requisite, as local bleedings can be repeated as often as necessary. The quantity to be abstracted in this way may vary from six to twelve ounces at a time, and the glasses should be applied just above the fissure between the nates. If it be judged proper to bleed from the lower part of the abdomen, leeches should be scattered above the pubes, from one groin to the other. In the progress of the complaint leeches may be applied to the labia, or even within the vestibulum, "by means of which more relief is sometimes obtained than by their application to the pubis." Local blood-letting should be employed once in three weeks or a month—provided the patient be not weakened by it, or exhausted by pain or discharge. Spontaneous hæmorrhage, to the extent of syncope, not unfrequently arrests for some time the progress of the disease. Even in the latter stages, when the loss of blood might appear unwarrantable, it may still be proper to recommend it; since it is well known that carcinoma uteri, in its ulcerative stage, involves all the neighbouring parts in a state of inflammation and irritation. For instance, when the rectum is attacked, there is tenesmus, great heat in that part, increased distress in voiding the fæces, exquisite tenderness of the gut, if the finger be carried into it. So in like manner, if the disease proceeds to the bladder, shivering usually comes on, succeeded by great pain, and strangury. If the disease makes its way, which is not very common, into the abdomen, symptoms of peritoneal inflammation will, of course, make their appearance. All these circumstances, therefore, call for the loss of blood, even in a late period of the disease.

The management of the discharge is a matter of great importance; for there is reason to believe that the spreading of carcinomatous ulcerations may be greatly retarded by the absorption or removal of the ichorous fluid secreted by them.

"Of all the modes of applying water to sores at the upper part of the vagina, none is so effectual as the use of the hip-bath; in the employment of which, the water is brought into contact with the sore without any risk of injuring the latter. By these means, the object of maintaining cleanliness is not only obtained, but a soothing application is made to an irritable surface; the careful injection of warm water into the vagina, by a syringe, or the agitation of the water by the hand, will render it more likely to remove any portions of coagulating lymph or thickened matter which may adhere to the inside of the vagina. The heat of the water employed should depend upon the feelings of the patient in some measure; but, generally speaking, it may vary from about 86° to 94°. Where the patient is too weak to bear the exertion of being placed in a hip-bath, her hips may be brought over the edge of the bed, and warm water may be carefully injected into the vagina by a female syringe. The quantity of the discharge is frequently increased by the means above-mentioned, but the comfort which the patient will derive from it will abundantly compensate her for any debility which may be produced by the remedy; and excruciating attacks of pain are sometimes rendered very sufferable by a frequent recurrence to it. Strong decoction of carrots, sometimes used for the same purpose, has the happiest effects. Warm water may also be made the vehicle for a variety of sedative applications, which are found by experience to tranquillize all irritable sores; and, in some, to expedite the healing process. Amongst the different applications for this purpose, the extractum conii, or extractum hyoscyami, may be mentioned, either of which may be employed in the proportion of about three or four drachms to a pint of water. Solutions of opium, or of extract of poppy, may also be used; of the former, two drachms; of the latter, half an ounce, may be dissolved in each pint of water. Starch, or mucilage of quince-seed, form good menstrua for these applications; their adhesive property enabling them to cling to surfaces to which they are applied. Three or four ounces of either of these fluids, impregnated with sedative substances, may be thrown into the rectum in those cases where relief is not obtained by their application to the vagina; but when opium is used for this purpose, the practitioner should be very careful to watch over its effects, as it has sometimes happened that unpleasant consequences have arisen from the application of this drug to the rectum, such as vomiting, syncope, cold extremities, and irregularity of the circulation. The action of the absorbents of the rectum is, in all probability, in these cases, increased by the inflammatory process which exists in the vicinity; besides which, the action of the rectum itself is temporarily taken off, so that the enema will probably be retained during a considerable length of time. Plasters and liniments, into the composition of which opium enters largely, will sometimes be found serviceable in allaying pain, and are useful auxiliaries in a disease in which all the resources of the practitioner may be required to diminish the sufferings of the patient." 229.

A dilute mixture of acetic or nitric acid in water, forms a soothing application to an irritable part, as acidi acetici ℥ss. aquæ distillatæ oj. m. fiat injectio. Or, acidi nitrici gutt. x. aquæ distillatæ oj. m. ft. injectio. Some liquor plumbi acetatis may be added to either of the above. If the discharge become so profuse as to cause debility, astringent injections must be employed: as decoct. cort. granati oj. sulphatis aluminis ℥ss. m. ft. injectio. Or, half a drachm of sulphate of zinc, fifteen ounces of water, and an ounce of tincture of kino, make a useful injection. If hæmorrhage occur, and the patient be in great pain at the time, it may be right *not* to restrain it hastily, unless the patient's strength has been previously much exhausted. When it is desirable to restrain the flow of blood, the foregoing injections may suffice; or ten grains of argenti nitras to a pint of distilled water.

As to internal remedies, pain being the symptom most complained of by the patient, the sedative class of medicines form our sheet anchor. But, as our author justly observes, we should never exhibit a medicine of strong sedative power, when a milder will produce equal relief—since the disease is of long duration, and every medicine will at last fail in producing its effect. Hyoscyamus and conium may be first employed, and afterwards we must have recourse to stramonium, belladonna, and opium. In some constitutions the solid opium, in others the liquid form will agree best—some prefer liq. opii sedativus—others, the black drop—in short, he will be the best practitioner who best understands the adaptation of these means to their end, and who can smooth the passage of the afflicted patient from this to another, and, we hope, a better world!

We have now turned over the last page of Mr. Clarke's work, and take our leave of him, perhaps for ever. We implicitly believe the concluding assertion.

> "In thus concluding his work, the writer can conscientiously assert, that he has made no statements which, in his opinion, are not founded in fact, and that he has withheld nothing which might in any way tend to the advantage of the practitioner, or to the comfort of the patient." 237.

The language is plain and perspicuous, and Mr. Clarke has the art of embodying his ideas in the most distinct and tangible shapes—an art, of all others, the most useful and difficult. To us, who are plain practical men, the work appears highly valuable; and we leave it to our readers to form their own conclusions from the ample specimens which we have now laid before them.

